# Enhancing Digital Breast Tomosynthesis Sinograms via Budget-Constrained PSO–Nelder–Mead: A Vision Transformer Assessment

**DOI:** 10.3390/biomimetics11080586

**Published:** 2026-08-17

**Authors:** Luis Fernando Rosas-Ordaz, Estefania Ruiz-Muñoz, Saúl Zapotecas-Martínez, Leopoldo Altamirano-Robles, Raquel Díaz-Hernández, José de Jesús Velázquez Arreola

**Affiliations:** 1 Instituto Nacional de Astrofísica Óptica y Electrónica, Sta. María Tonantzintla, Puebla 72840, Mexico; estefania.ruiz@inaoep.mx (E.R.-M.); robles@inaoep.mx (L.A.-R.); raqueld@inaoep.mx (R.D.-H.); josej.velazqueza@inaoep.mx (J.d.J.V.A.); 2 Secretaría de Ciencia, Humanidades, Tecnología e Innovación (SECIHTI), Mexico City 03940, Mexico

**Keywords:** hybrid algorithms, Particle Swarm Optimization, contrast enhancement, sinograms, tomosynthesis

## Abstract

Sinogram images represent projection-domain data acquired during digital breast tomosynthesis (DBT), preserving angular information prior to reconstruction. However, their low contrast and noise-related degradation limit their direct use in downstream tasks. This work proposes a budget-constrained hybrid biomimetic optimization framework for projection-domain contrast enhancement based on the integration of Particle Swarm Optimization (PSO) and the Nelder–Mead (NM) simplex method. The approach combines global exploration with local refinement under a fixed number of objective function evaluations (NFE), enabling fair and computationally efficient comparisons with standalone optimizers. Experiments were conducted on 222 labeled mammographic images (136 benign and 86 malignant), which were transformed into sinograms via the Radon transform. The proposed method achieves competitive performance in terms of PSNR, SSIM, and FSIM, while exhibiting faster convergence and reduced computational cost compared to several state-of-the-art swarm-based approaches. Additionally, the impact of the enhanced sinograms was evaluated through a downstream classification task using a Vision Transformer-based knowledge distillation scheme, demonstrating improved discrimination between benign and malignant cases. These results demonstrate that the proposed budget-constrained hybrid biomimetic strategy provides an effective and computationally efficient solution for contrast enhancement in projection-domain medical imaging.

## 1. Introduction

Digital Breast Tomosynthesis (DBT) has become a widely adopted imaging modality for breast cancer detection due to its ability to reduce tissue overlap and improve lesion conspicuity compared to conventional mammography [1]. During DBT acquisition, multiple X-ray projections are collected and represented as sinograms, which encode the raw projection data prior to image reconstruction. Unlike reconstructed images, sinograms preserve angular and projection-domain information that may be partially lost during reconstruction, making them a valuable yet underutilized source for breast lesion analysis [2,3].

Despite containing complete acquisition information, sinogram images are inherently affected by low contrast, noise, and acquisition-related artifacts, which severely limit their interpretability and direct applicability to diagnostic tasks. These limitations are particularly critical in dense breast tissue, where subtle lesions may remain obscured.

Image enhancement is an important preprocessing step for computer vision and medical imaging applications. Images often fail to clearly show subtle details caused by inadequate lighting or sensor limitations. Although histogram-based methods like Histogram Equalization (HE) and its variants are computationally efficient, they often introduce artifacts or unnatural appearances and can amplify noise [4,5]. Thus, the suboptimal contrast in sinogram images represents a major obstacle to direct use in breast lesion detection and classification. Current enhancement strategies often struggle to handle the high variability and nonlinearity of projection-domain data, limiting the clinical applicability of sinogram-based analysis. Consequently, image enhancement has increasingly been formulated as an optimization problem in which metaheuristic algorithms maximize objective functions based on entropy or edge information.

Biomimetic optimization provides a biologically inspired paradigm for solving complex optimization problems. Bio-inspired optimization algorithms emulate collective or evolutionary behaviors observed in natural systems and have demonstrated strong performance in nonlinear, high-dimensional search spaces without requiring large, annotated datasets. Biomimetic optimization leverages principles such as decentralized intelligence and self-organization to navigate rugged fitness landscapes, much as biological populations adapt to environmental pressures. Representative examples include Particle Swarm Optimization (PSO) [6], Standard PSO (SPSO), Constriction PSO, Genetic Algorithms (GA) [7], Artificial Bee Colony (ABC) [8], and Differential Evolution (DE) [9] which draw inspiration from swarm intelligence, natural selection, and foraging behavior. These algorithms are representative bio-inspired approaches that have been successfully applied to tasks including medical image enhancement, contrast optimization, and noise suppression [5,10].

Among the metaheuristics mentioned above, PSO is prominent due to its ease of implementation and ability to explore complex search spaces. However, PSO frequently suffers from premature convergence and a lack of fine-tuning capabilities; it often oscillates around promising regions and does not reach local optima. To overcome these limitations, this study presents a hybrid optimization framework that combines global exploration capabilities of PSO with the local refinement capabilities of the Nelder–Mead (NM) simplex search method. In particular, a budget-constrained optimization strategy is used to balance global exploration and local refinement under a fixed computational budget constraint.

The adoption of a fixed Number of Function Evaluations (NFE) enables fair comparison between optimization algorithms while also constraining computational resources. Combining the global exploration capabilities of swarm intelligence with the deterministic local refinement of NM enables exploitation within promising regions and mitigates stagnation effects commonly observed in standalone swarm-based methods.

Bio-inspired methods offer an attractive alternative when labeled projection-domain datasets remain limited, as they rely on fitness-driven global search strategies rather than large quantities of annotated data. Unlike deep learning techniques, which may suffer from overfitting when trained on small amounts of training data [11] and may exhibit sensitivity to initialization or training dynamics [12], bio-inspired optimization algorithms can operate directly on image quality objectives without the need for supervised labels. This property makes them particularly suitable for sinogram analysis where labeled datasets remain limited and reconstruction-free processing is desirable. In this study, the impact of the proposed enhancement method on breast lesion discrimination is assessed using a downstream evaluation strategy based on a Vision Transformer-based classification framework.

The primary objective of this study is to systematically compare multiple bio-inspired optimization algorithms, including PSO and its variants (SPSO, Constriction PSO), GA, ABC, and DE for contrast enhancement of sinogram images. Furthermore, a Budget-Constrained Particle Swarm Optimization–Nelder–Mead (BC-PSO-NM) algorithm is proposed to accelerate convergence and improve solution quality. Lastly, this study examines how contrast enhancement influences breast lesion classification performance.

The main contributions of this work are summarized as follows:

- A projection-domain contrast enhancement framework for DBT sinograms based on bio-inspired optimization algorithms, enabling reconstruction-free image enhancement.
- The development of a Budget-Constrained Hybrid Particle Swarm Optimization–Nelder–Mead algorithm, in which the transition between global exploration and local simplex refinement is governed by a fixed NFE, enabling deterministic allocation of computational resources and fair comparison across bio-inspired optimizers.
- A statistically validated comparative analysis (Wilcoxon signed-rank test, α=0.05) demonstrating robustness and reproducibility across multiple runs.
- A comprehensive quantitative evaluation using PSNR, SSIM, and FSIM, together with computational efficiency analysis under strict evaluation budgets.
- An experimental validation showing that contrast-optimized sinograms significantly improve breast lesion classification performance, supporting the potential clinical relevance of projection-domain processing.

The results highlight the effectiveness of the proposed BC-PSO-NM method in improving convergence behavior and optimization stability while demonstrating that enhanced sinograms contribute to better lesion discrimination. Furthermore, the enhanced sinograms lead to measurable improvements in downstream benign–malignant classification performance, indicating that projection-domain contrast enhancement can positively influence diagnostic discrimination. These findings reinforce the potential of projection-domain processing as a complementary approach for medical image analysis.

The remainder of this paper is organized as follows. Section 2 reviews related work on the use of sinograms, contrast enhancement using traditional techniques, and bio-inspired optimization algorithms. Section 3 describes the proposed bio-inspired optimization framework and contrast enhancement methods. Section 4 details the experimental setup, fitness functions, and evaluation metrics. Section 5 presents quantitative and qualitative results, including an analysis of execution times and classification performance. Section 6 discusses the implications of the findings within the context of biomimetics and medical imaging. Finally, Section 7 concludes the paper and outlines future research directions.

## 2. Related Works

There has been an increasing interest in analyzing and processing sinograms for medical imaging since they contain all the original acquisition information before it is reconstructed into an image. Sinograms provide projection-domain properties that can help with noise analysis, contrast improvement, tumor location, and classification. As a result, there have been recent studies exploring different ways to apply deep learning and optimization methods that operate directly in the sinogram domain, thereby mitigating information loss during reconstruction.

### 2.1. Sinogram-Based Analysis in Medical Imaging

Recent studies have demonstrated that sinogram data can support diagnostic analysis without requiring image reconstruction. For example, Sindhura et al. [13] presented a deep learning method that automatically detects and classifies intracranial hemorrhages from sinograms, achieving improvements of up to 27% in patient-wise accuracy compared with state-of-the-art Computed Tomography (CT)-based approaches. Similarly, Yoon et al. [14] applied a transformer model to sinogram patterns for segmentation in limited-angle CT, achieving Dice Similarity Coefficient (DSC) values of 56.71% and 66.14% on the CTICH and FUMPE datasets, respectively, at 30°, compared with 52.90% and 60.57% obtained by the best reconstruction-based methods.

Furthermore, Lu et al. [15] evaluated a physics-informed sinogram completion method for reducing metal artifacts caused by neurovascular coils in brain CT images. Their study compared the proposed approach with traditional and deep learning-based metal artifact reduction methods, demonstrating the relevance of projection-domain processing for preserving diagnostic information and limiting newly introduced artifacts.

In the field of breast imaging, Ruiz-Muñoz et al. [3] investigated the use of mammographic and DBT sinograms for lesion classification, reporting accuracy, recall, and F1-score values of 94.96%, thereby supporting the effectiveness of projection-domain representations for discriminating between benign and malignant lesions without requiring full image reconstruction. Their results showed that sinograms preserve diagnostically relevant information that is often redistributed or attenuated during reconstruction.

Subsequent studies showed that sinogram-based approaches can outperform image-based approaches. For example, ResNet18 achieved an accuracy of 98.53% using sinograms generated from 180° projections, compared with 93.49% using images reconstructed through simple backprojection [2]. Collectively, these studies demonstrate that the sinogram domain preserves spatial and structural information that can be harnessed by advanced learning models to enhance diagnostic performance.

### 2.2. Traditional and Deep Learning-Based Contrast Enhancement Techniques

Traditional contrast enhancement techniques applied to projections have generally been linear and non-linear transforms, along with histogram-based techniques. Although simple, these methods rarely produced good results in the presence of noise and projection inhomogeneity.

Adaptive and learning-based approaches, such as deep-learning-based sinogram reconstruction, have also shown promise. In fact, a deep learning framework for PET image reconstruction directly from the sinogram domain achieved an SSIM close to 0.94 and a mean relative RMSE of approximately 2.74, demonstrating its ability to generate high-quality reconstructed images [16].

### 2.3. Bio-Inspired Optimization for Image Enhancement

Nature-inspired optimization algorithms have been studied for medical image enhancement applications, including thresholding, segmentation, and contrast optimization in image processing. Representative approaches include PSO, ABC, DE, and GA, which continue to demonstrate competitive performance in noisy and ill-posed medical imaging problems [17,18].

Recently, Rosas-Ordaz et al. [5] presented a bio-inspired framework for enhancing tomosynthesis sinogram contrast through PSO. Their results showed that PSO-based enhancement achieved a PSNR of 15.41 dB and an SSIM of 0.76, compared with 12.96 dB and 0.70 obtained using linear intensity adjustment, respectively. These findings further underscore the potential applicability of swarm-based optimization algorithms for sinogram analysis under conditions involving noise and acquisition-related artifacts.

Although Genetic Algorithms are among the earliest evolutionary optimization techniques, they remain relevant in hybrid contrast enhancement frameworks and multi-objective optimization schemes, often achieving competitive performance when combined with adaptive fitness functions or morphological constraints [19].

### 2.4. Hybrid Bio-Inspired Algorithms

Hybrid bio-inspired algorithms can be defined as the use of at least one population-based bio-inspired optimizer along with a complementary search method (such as a local deterministic search or gradient-based refinement). The bio-inspired component provides global exploration capabilities by discovering areas of interest within the search space, whereas the second component performs local refinement on promising candidates [20]. When each of the components operates in consecutive stages, it is classified as a hybrid sequential metaheuristic according to Talbi’s taxonomy [21]. This type of exploration–exploitation balance has been shown to increase convergence speeds and robustness in complex optimization problems.

The use of hybrid bio-inspired methods in medical imaging applications has shown great robustness when dealing with noisy data and high-dimensional conditions [22]. For example, Surbhi Vijh et al. [23] proposed a hybrid WOA-APSO system to identify an optimal feature subset in lung CT images, followed by a CNN for automatic lung tumor classification. Similarly, for medical image segmentation and image fusion tasks, Gu et al. [24] proposed a multi-stage hybrid optimization method for 3D medical image segmentation, achieving similarity indices of 0.963, 0.956, and 0.978 on the CJH27, heart, and kidney datasets, respectively, with an average value of 0.966. Hybrid methods such as Genetic-Grey Wolf Optimization have been used in medical image fusion to improve structural information preservation and contrast [25].

However, despite their promising performance, many hybrid studies do not explicitly enforce standardized computational constraints, such as equal NFE budgets, which may limit fairness in comparative performance assessments. Furthermore, their application to projection-domain contrast enhancement in DBT sinograms remains largely unexplored.

### 2.5. Research Gap and Contribution of This Work

Despite the growing body of sinogram-based deep learning research, comparative studies that systematically evaluate multiple bio-inspired optimization algorithms for direct sinogram contrast enhancement—and subsequently assess their impact on lesion classification performance—remain scarce.

Most existing works focus either on image reconstruction or on a single optimization technique, often without enforcing standardized computational constraints across methods. As a result, reported performance gains may be influenced by unequal evaluation budgets rather than intrinsic algorithmic efficiency.

To address this limitation, this study presents a systematic comparison of several bio-inspired algorithms (PSO, SPSO, Constriction PSO, and ABC) and evolutionary approaches (DE and GA) for projection-domain contrast enhancement under a shared NFE budget. In addition, a Budget-Constrained Hybrid Particle Swarm Optimization–Nelder–Mead (BC-PSO-NM) algorithm is introduced to accelerate convergence while preserving identical computational resources across all methods.

Although hybrid metaheuristics combining Particle Swarm Optimization with local search strategies such as the Nelder–Mead method have been explored in general optimization problems, their application specifically to projection-domain contrast enhancement in DBT remains largely unexplored. From a theoretical optimization perspective, the No Free Lunch theorem suggests that optimization performance is inherently problem-dependent, implying that algorithms successful in one domain may not necessarily exhibit the same behavior in another [26]. Consequently, investigating specialized optimization frameworks for projection-domain medical imaging constitutes a relevant research direction. This observation motivates the evaluation and development of tailored hybrid strategies for DBT sinogram enhancement.

Finally, the enhanced sinograms are evaluated in a downstream lesion classification task, establishing a direct link between optimization-based contrast enhancement and diagnostic performance. This integrated analysis contributes both to projection-domain medical image processing research and to methodological rigor in comparative metaheuristic evaluation, while also highlighting the potential clinical feasibility of optimized sinogram-based diagnosis.

## 3. Theoretical Background

This section presents the theoretical foundations underlying the proposed framework. First, the projection-domain representation of digital tomosynthesis through sinograms is introduced, together with the noise and contrast degradation characteristics that motivate enhancement in this domain. Subsequently, the principles of projection-domain contrast enhancement, bio-inspired optimization, and Nelder–Mead local search are described, along with the fitness function employed to guide the optimization process. Finally, the image quality assessment metrics, statistical analysis procedures, and the relationship between contrast enhancement and downstream lesion classification are discussed.

### 3.1. Sinogram Representation in Medical Imaging

In X-ray-based imaging systems such as computed tomography, digital mammography, and DBT, the acquired data are fundamentally represented in the projection domain as sinograms. A sinogram corresponds to the Radon transform of an object, where each point represents the line integral of X-ray attenuation coefficients along a specific projection angle and detector position.

Formally, for a two-dimensional object f(x,y), the Radon transform is defined as:

(1) R(θ,s)=∫∫f(x,y)δ(xcosθ+ysinθ−s)dxdy,

where θ denotes the projection angle, δ(·) denotes the Dirac delta function and *s* represents the detector coordinate [27].

Unlike reconstructed images, sinograms preserve raw acquisition information, including angular continuity and projection-domain patterns. In breast imaging, lesions generate consistent trajectories across projection angles, which may remain detectable in sinograms even when reconstruction artifacts, tissue overlap, or limited-angle geometry degrade their spatial-domain appearance [28]. This property has motivated recent research on direct sinogram analysis for lesion localization and classification, avoiding reconstruction-induced information loss [3].

### 3.2. Contrast Degradation and Noise Characteristics in Sinograms

Although sinograms contain rich acquisition information, they are inherently affected by low contrast and high noise levels, particularly in low-dose and limited-angle acquisitions such as DBT. As a result of differences in heterogeneous soft tissue attenuation through variable thicknesses of tissue, varying degrees of breast composition, and acquisition geometries, projection intensities vary stochastically and are therefore nonstationary and dependent upon the projection angle [29].

In low-dose applications, quantum or Poisson noise dominates because of stochastic fluctuations that propagate across projection angles. In contrast to reconstructed images, where one may filter out some portion of noise, sinogram noise is inherently present within projection-domain signals and has the potential to mask lesion-related features [30]. Due to these two limiting factors, the class separability is greatly diminished, and both visual evaluation and automatic assessment are inhibited.

Conventional methods for manipulating projection-domain intensities may amplify noise already present in the sinogram, which can be further propagated during image reconstruction [31]. This motivates the need for adaptive techniques designed to improve contrast while maintaining the structural integrity of the projections.

### 3.3. Contrast Enhancement in the Projection Domain

Contrast enhancement in sinograms differs fundamentally from spatial-domain enhancement. Sinogram intensities represent line integrals along individual rays rather than local pixel brightness. Therefore, enhancement strategies must preserve angular consistency and avoid introducing artificial discontinuities into the projection data.

Recent studies have reformulated sinogram contrast enhancement as an optimization problem. In this formulation, transformation parameters are adaptively determined by maximizing multiple objective criteria. Representative objective functions include information-based measures such as entropy, which quantifies global image information, and edge-based measures such as the Sum of Edge Intensity and the Count of Edge Pixels, which promote structural detail preservation while limiting noise amplification [5].

### 3.4. Bio-Inspired Optimization Principles

Bio-inspired optimization methods that model the biological processes of evolution and collective intelligence can provide powerful means to solve many difficult and nonlinear stochastic problems using no gradient information. As derivative-free methods, they are particularly relevant to projection-domain contrast enhancement, where the objective function may be multimodal, non-differentiable, and computationally expensive.

Particle Swarm Optimization models the flocking or schooling behavior of birds and fish while foraging. A population of candidate solutions called particles moves through the search space by combining their own experience with the swarm’s collective knowledge. The dynamics of each particle *i* are governed by the velocity and position update equations:

(2) $$v_{i}\left(t+1\right)=v_{i}\left(t\right)+c_{1}r_{1}\left(P_{best,i}\left(t\right)-x_{i}\left(t\right)\right)+c_{2}r_{2}\left(G_{best}\left(t\right)-x_{i}\left(t\right)\right)$$

(3) $$x_{i}\left(t+1\right)=x_{i}\left(t\right)+v_{i}\left(t+1\right)$$

where $c_{1}$ and $c_{2}$ represent acceleration coefficients, $r_{1}$ and $r_{2}$ are random vectors uniformly distributed in (0,1), $P_{best,i}\left(t\right)$ is the personal best position of particle *i*, and $G_{best}\left(t\right)$ denotes the global best position found by the entire swarm. Modern variants, such as Standard PSO and Constriction PSO, are extensions of this dynamic model that add an inertial weight (ω) or constriction factor (χ) to increase the stability and speed of convergence. Additionally, PSO is one of the most popular methods for balancing exploration and exploitation in medical image enhancement tasks [32].

Other swarm intelligence methods include Artificial Bee Colony [33], which dynamically allocates search effort by modeling bee-foraging behavior and can effectively process images with non-uniform intensity distributions. Evolutionary algorithms (EAs), including Genetic Algorithms and Differential Evolution, constitute another relevant family of optimization methods. The use of selection, mutation, crossover, and recombination operators within EAs allows them to explore complex fitness spaces. Recent studies indicate that EAs can achieve rapid convergence in continuous optimization problems involving noisy medical imaging data [34].

### 3.5. Nelder–Mead Simplex Search

The Nelder–Mead simplex method operates on a simplex composed of n+1 vertices in an *n*-dimensional search space [35]. At each iteration, the vertices are ordered according to their objective function values, and the worst-performing point is replaced through a sequence of geometric transformations designed to explore or contract the search region adaptively.

The reflection operation attempts to explore the search space by moving the worst vertex away from the centroid of the remaining points. It is defined as:

(4) $$x_{r}=x_{c}+\alpha \left(x_{c}-x_{n+1}\right),$$

where α>0 is the reflection coefficient. Reflection allows the simplex to probe unexplored regions while preserving its geometric structure.

If the reflected point significantly improves the objective function, an expansion step is performed to accelerate movement in promising directions:

(5) $$x_{e}=x_{c}+\gamma \left(x_{r}-x_{c}\right),$$

where γ>1 is the expansion coefficient. Expansion increases the step size toward regions of rapid improvement.

When reflection does not yield sufficient improvement, a contraction step is applied to reduce the simplex size and focus the search locally:

(6) $$x_{con}=x_{c}+\rho \left(x_{n+1}-x_{c}\right),$$

where 0<ρ<1 is the contraction coefficient. This operation allows the simplex to retreat toward more promising regions.

If contraction also fails to improve the objective function, a shrink operation is executed, reducing the entire simplex toward the best vertex:

(7) $$x_{i}=x_{1}+\sigma \left(x_{i}-x_{1}\right),\;i=2,\ldots ,n+1,$$

where 0<σ<1 is the shrink coefficient. The shrink step reduces the search region, improving local refinement and preventing oscillatory behavior.

All transformations are performed relative to the centroid of the simplex, excluding the worst vertex, defined as:

(8) $$x_{c}=\frac{1}{n}\sum_{i=1}^{n}x_{i},\;x_{i}\neq x_{n+1}$$

This centroid-based geometric adaptation enables derivative-free local refinement with low computational overhead, making the Nelder–Mead method particularly suitable as an exploitation stage within hybrid optimization frameworks [36].

### 3.6. Fitness Functions for Contrast Enhancement

The effectiveness of bio-inspired optimization critically depends on the fitness function, which defines enhancement quality. In sinogram enhancement, fitness functions should emphasize information content and structural preservation rather than pixel-wise fidelity.

In contrast enhancement using metaheuristic optimization, there is currently no universally accepted or standardized fitness function. Consequently, many studies rely on composite objective functions that combine complementary image quality indicators such as entropy, edge information, and structural detail preservation.

One of the most widely adopted formulations was originally introduced by Munteanu and Rosa [37], and later employed in metaheuristic-based enhancement frameworks such as Khan et al. [10]. This objective function provides a balanced assessment of contrast enhancement by integrating entropy-based and edge-based criteria. Entropy (EI) is widely adopted in image enhancement tasks, as increased entropy is generally associated with improved dynamic range utilization and enhanced visibility of diagnostically relevant structures. To further capture structural information, edge-based metrics such as the Sum of Edge Intensity (SEI) and the Count of Edge Pixels (CEP) are integrated into the objective formulation. These measures provide complementary structural information by emphasizing edge strength and spatial detail, enabling a more comprehensive assessment of contrast enhancement that balances global intensity distribution and local structural preservation. Alternative fitness functions based exclusively on entropy or edge information have also been reported in the literature; however, such formulations may emphasize only a subset of image characteristics. The logarithmic transformation applied to the SEI term reduces the influence of large edge-intensity variations, preventing the optimization process from being dominated by extreme responses. Furthermore, recent studies have demonstrated that this fitness formulation is effective for projection-domain contrast enhancement, yielding consistent qualitative and quantitative improvements in sinogram analysis [5]. The resulting fitness function is defined in Equation (9).

(9) $$F\left(I_{E}\right)=\log\left(\log\left(SEI\right)\right)\times CEP\times EI$$

### 3.7. Image Quality Assessment and Statistical Analysis

To quantitatively evaluate contrast enhancement performance, widely adopted full-reference image quality metrics are employed, including: Peak Signal-to-Noise Ratio, Structural Similarity Index Measure, and Feature Similarity Index.

Peak Signal-to-Noise Ratio (PSNR) measures the fidelity between the original and enhanced images based on the Root Mean Squared Error (RMSE) [38]. It is defined as:

(10) $$PSNR=20\log_{10}\left(\frac{255}{RMSE}\right)$$

such that

(11) $$RMSE=\sqrt{\frac{1}{R_{o}C_{o}}\sum_{i=1}^{R_{o}}\sum_{j=1}^{C_{o}}{\left(I_{o}\left(i,j\right)-I_{e}\left(i,j\right)\right)}^{2}}$$

where, $R_{o}$ and $C_{o}$ denote the image dimensions, $I_{o}$ represents the original image, and $I_{e}$ corresponds to the enhanced image. Higher PSNR values indicate smaller reconstruction error.

Structural Similarity Index Measure (SSIM) evaluates perceptual similarity by jointly considering luminance, contrast, and structural components [39]. It is defined as:

(12) $$SSIM=\frac{\left(2\mu_{o}\mu_{e}+C_{1}\right)\left(2\sigma_{oe}+C_{2}\right)}{\left(\mu_{o}^{2}+\mu_{e}^{2}+C_{1}\right)\left(\sigma_{o}^{2}+\sigma_{e}^{2}+C_{2}\right)}$$

where $\mu_{o}$ and $\mu_{e}$ denote mean intensities, $\sigma_{o}^{2}$ and $\sigma_{e}^{2}$ represent variances, $\sigma_{oe}$ is the covariance between the original and enhanced images, and $C_{1}$ and $C_{2}$ are stabilization constants. Values closer to 1 indicate a higher degree of structural preservation.

Feature Similarity Index (FSIM) evaluates similarity based on perceptually significant features, specifically Phase Congruence (PC) and Gradient Magnitude (GM) [40]. The local similarity components are defined as:

(13) $$S_{PC}\left(x\right)=\frac{2PC_{1}\left(x\right)PC_{2}\left(x\right)+T_{1}}{PC_{1}{\left(x\right)}^{2}+PC_{2}{\left(x\right)}^{2}+T_{1}}$$

(14) $$S_{GM}\left(x\right)=\frac{2GM_{1}\left(x\right)GM_{2}\left(x\right)+T_{2}}{GM_{1}{\left(x\right)}^{2}+GM_{2}{\left(x\right)}^{2}+T_{2}}$$

The overall FSIM is computed by combining these local similarities weighted by the maximum phase congruence:

(15) $$FSIM=\frac{\sum_{x\in \Omega }S_{PC}\left(x\right)S_{GM}\left(x\right)PC_{m}\left(x\right)}{\sum_{x\in \Omega }PC_{m}\left(x\right)}$$

where $PC_{m}\left(x\right)=\max\left(PC_{1}\left(x\right),PC_{2}\left(x\right)\right)$, and $T_{1}$ and $T_{2}$ are small positive constants. FSIM ranges from 0 to 1, with values closer to 1 indicating greater similarity.

To assess the statistical significance of the performance differences between algorithms, the Wilcoxon signed-rank test is useful [41]. This non-parametric test evaluates paired samples without assuming that the data follow a normal distribution, making it particularly suitable for optimization results obtained across multiple independent runs or image instances. The test determines whether the median difference between two methods is statistically significant.

### 3.8. From Contrast Enhancement to Lesion Classification

Contrast improvement is useful only to the extent that it enhances the ability to make a diagnosis. The ability to distinguish between features within a sinogram depends on the angular coherence of the pattern that encodes the presence or absence of a lesion. Recent studies have shown that improving projection-domain image quality before classification can enhance discrimination between benign and malignant lesions [2,3]. Furthermore, improving image quality while preserving angular structure can reduce the adverse effects of random noise and acquisition-related artifacts on the discriminative information contained in sinograms. This theoretical linkage justifies evaluating contrast enhancement not only through image quality metrics but also through classification performance, forming the conceptual basis of the bio-inspired optimization framework proposed in this work.

## 4. Methodology

**Figure 1:**
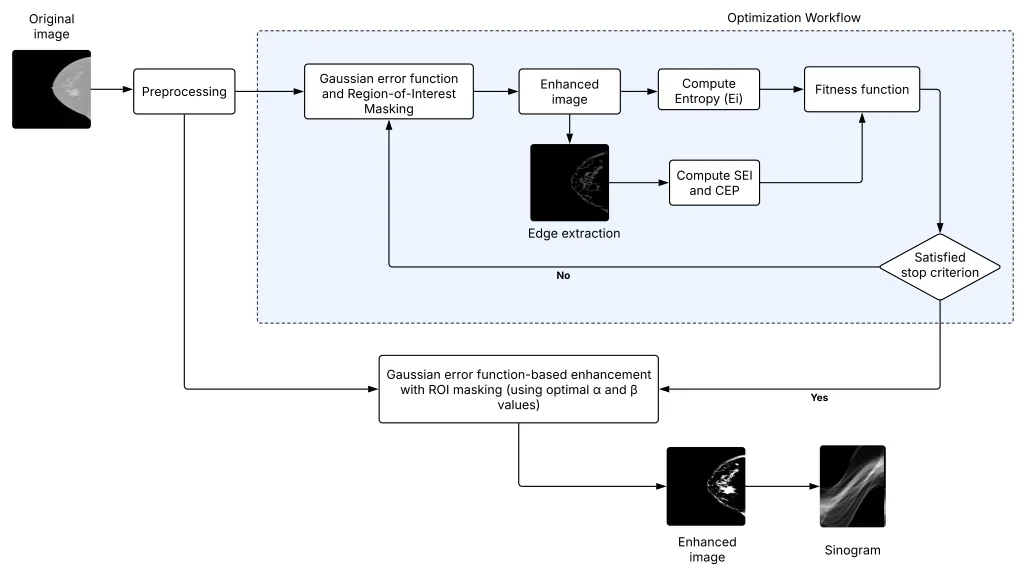
Methodology for improving contrast and generating sinograms.

This section presents the proposed framework for contrast enhancement and classification of tomosynthesis images in the projection domain. The methodology follows a structured pipeline comprising: (i) dataset characterization; (ii) preprocessing of mammographic images; (iii) formulation of the parametric contrast enhancement model; (iv) optimization of the model parameters using multiple bio-inspired algorithms, including the proposed BC-PSO-NM algorithm; (v) generation of sinograms via the Radon transform; and (vi) classification of enhanced sinograms into benign and malignant categories. The contrast enhancement workflow and sinogram generation are illustrated in Figure 1.

### 4.1. Dataset Description

Currently, publicly available breast imaging datasets do not provide annotated sinogram representations. Therefore, breast tomosynthesis images were employed as the primary source data to allow controlled generation of projection-domain representations while preserving diagnostic labels. This study was conducted using DBT [42]. A total of 222 labeled DBT images were included, comprising 136 benign and 86 malignant cases. These annotated samples enabled both quantitative contrast enhancement evaluation and downstream lesion classification analysis. Although the resulting class distribution is moderately imbalanced, the sample size and class composition reflect the scarcity of publicly available labeled breast projection-domain data.

Since existing datasets provide annotations only in the spatial domain, sinograms were generated to enable projection-domain analysis while maintaining the original diagnostic labels. All experiments were conducted using standardized preprocessing and a fixed projection acquisition configuration to ensure reproducibility.

After contrast enhancement, sinogram images were obtained from the optimized DBT images using a simulated Radon projection process, as detailed in Section 4.5. This strategy ensures consistency between spatial-domain labels and projection-domain analysis.

### 4.2. Preprocessing

Prior to contrast enhancement, the digital mammograms underwent a standardized preprocessing procedure to ensure consistency across samples while preserving structural information. The preprocessing steps included:

- Image dimensions: The mammographic images were maintained at a spatial resolution of 640×640 pixels to ensure consistent input dimensions.
- Intensity representation: Pixel intensities were represented within the 8-bit range [0,255].
- Noise smoothing: To reduce high-frequency fluctuations while preserving anatomical structures, a median filter was applied.
- Tissue Adjustment: A region of interest was defined using the intensity interval [100,255]. Pixels outside this region were attenuated by subtracting 83, corresponding to the mean intensity estimated from a representative tissue region.

These preprocessing operations were applied consistently across all images to reduce background variations and standardize the input data before optimization while preserving relevant anatomical structures.

### 4.3. Contrast Enhancement Model

Contrast enhancement is formulated as a parametric intensity transformation based on the Gauss error function. The formulation follows the adaptation proposed by Pérez-Enríquez et al. [43]. The enhanced intensity $I_{E}\left(x\right)$ is defined as:

(16) $$I_{E}\left(x\right)=\frac{\Delta }{2}\left[1+\frac{2}{\sqrt{\pi }}\int_{0}^{\frac{x-\beta }{\alpha }}e^{-t^{2}}dt\right]$$

where *x* represents the pixel intensities, Δ represents the image intensity levels (Δ=255 for 8-bit images), and α and β are the parameters to be optimized, subject to α>0 and β∈[0,Δ].

The Gauss error function is selected due to its smoothness, bounded output, and strictly increasing mapping, which preserves intensity ordering and enables controlled contrast stretching without introducing abrupt discontinuities. The search space is constrained to α∈[10,50] and $\beta \in [\frac{1}{3}\Delta ,\frac{2}{3}\Delta ]$ to ensure stable enhancement within the relevant intensity range.

To restrict enhancement only to the relevant regions, a Region of Interest (ROI) is defined based on intensity thresholds. Pixels within the range [100,255] are considered candidate lesion or tissue regions. Pixels outside this interval are attenuated by subtracting 83, corresponding to the rounded mean intensity estimated from a representative tissue region. Contrast enhancement is then applied selectively within the ROI, while the attenuated image content is preserved outside the ROI.

The optimal parameters (α,β) are determined through bio-inspired optimization using the fitness formulation described in the next subsection.

### 4.4. Optimization Framework

This subsection describes the general optimization strategy adopted to determine the optimal parameters of the proposed contrast enhancement model. The optimization process is formulated as a constrained parameter search problem under a fixed NFE budget to ensure fair comparison across algorithms. First, the general evaluation workflow is presented, followed by the proposed BC-PSO-NM algorithm and the configuration of the comparative bio-inspired algorithms.

#### 4.4.1. Optimization Workflow

The contrast enhancement problem is formulated as a parameter optimization task, where the objective is to determine the optimal values of (α,β) that maximize the fitness function defined in Equation (9).

For each candidate solution (α,β) generated by the optimization algorithm, the following evaluation procedure is performed:

1. Parametric Transformation: The Gauss error function transformation defined in Equation (16) is applied to the preprocessed image.
2. Region-of-Interest Masking: Contrast enhancement is selectively applied within the defined ROI to preserve non-target structures.
3. Feature Extraction: The entropy of the enhanced image is computed, and edge extraction is performed using binary thresholding followed by Canny filtering to obtain SEI and CEP metrics.
4. Fitness Evaluation: The fitness function used in this study follows the formulation proposed by Munteanu and Rosa [37]. This objective function combines EI, SEI, and CEP, as defined in Equation (9).

This evaluation cycle is repeated iteratively until the predefined stopping criterion is satisfied. In this study, convergence is controlled using a fixed NFE budget. The same total NFE budget is enforced for all algorithms to ensure a fair computational comparison. This design ensures that performance differences can be attributed to algorithmic characteristics rather than unequal computational effort.

Once the optimal parameters $\left(\alpha^{*},\beta^{*}\right)$ are obtained, the final enhanced image is generated by applying the same tissue attenuation and ROI masking procedure using the optimized parameter values. Pixels within the ROI are transformed using Equation (16) with $\left(\alpha^{*},\beta^{*}\right)$, while non-relevant regions retain their attenuated intensity values. This selective enhancement strategy ensures that contrast improvement is concentrated on diagnostically relevant structures.

#### 4.4.2. Budget-Constrained Hybrid Particle Swarm Optimization–Nelder–Mead Algorithm

To enhance convergence stability and accelerate parameter optimization, a hybrid BC-PSO-NM algorithm is proposed. According to Talbi’s taxonomy of hybrid metaheuristics [21], the proposed approach can be interpreted as a high-level relay hybrid metaheuristic. From a broader evolutionary computation perspective, it also belongs to the class of hybrid algorithms, which combine a population-based global search with an embedded local refinement mechanism. In this structure, two optimization algorithms are executed sequentially, with the output of the first method serving as the initialization for the second.

In the proposed framework, a fixed NFE budget is used as the primary resource constraint. Rather than allowing each optimizer to run independently, the available evaluations are sequentially allocated between PSO and NM. PSO first consumes a predefined portion of the budget to perform global exploration and identify promising regions of the search space. The best solutions obtained are then used to initialize a multi-start NM procedure, which exploits the remaining evaluations for local refinement. A best-so-far preservation mechanism is employed throughout the process, and the complete procedure is summarized in Algorithm 1.

To ensure reproducibility and transparent budget accounting, the PSO stage was configured with swarm size $N_{p}=20$, acceleration coefficients $c_{1}=1.7$ and $c_{2}=1.7$, and executed for $G_{\mathrm{PSO}}=12$ generations. The number of function evaluations consumed during the PSO phase is given by

$${\mathrm{NFE}}_{\mathrm{PSO}}=N_{p}\times G_{\mathrm{PSO}}.$$

The remaining evaluation budget is then assigned to the Nelder–Mead refinement stage:

$${\mathrm{NFE}}_{\mathrm{remaining}}={\mathrm{NFE}}_{\mathrm{total}}-{\mathrm{NFE}}_{\mathrm{PSO}}.$$

To promote local refinement from diverse regions of the search space, the global best solution together with the top-k=10 particles ranked according to their fitness values are selected as initialization points for the multi-start Nelder–Mead procedure. The value k=10 was empirically determined, providing a suitable compromise between refinement diversity and computational cost within the fixed evaluation budget.

For each starting point, an initial simplex is constructed using standard Nelder–Mead perturbations along each search dimension. The simplex then evolves through reflection, expansion, contraction, and shrink operations while respecting the remaining evaluation budget. The maximum number of evaluations assigned to each local search is given by

$${\mathrm{maxfev}}_{j}=\min\left({\mathrm{NFE}}_{\mathrm{remaining}},{\mathrm{maxfev}}_{\mathrm{start}}\right),$$

ensuring that the shared evaluation budget is never exceeded. In the experimental configuration used in this study, the PSO phase consumed

$${\mathrm{NFE}}_{\mathrm{PSO}}=N_{p}\times G_{\mathrm{PSO}}=20\times 12=240$$

leaving 200 evaluations for the Nelder–Mead refinement stage. Consequently, the total evaluation budget was fixed at

$${\mathrm{NFE}}_{\mathrm{total}}=440$$

The chosen distribution was determined empirically through preliminary experiments, providing a suitable balance between exploration capability and refinement efficiency under the fixed evaluation budget.
**Algorithm 1:** Budget-Constrained Hybrid PSO–NM (BC-PSO-NM) under a shared NFE budget
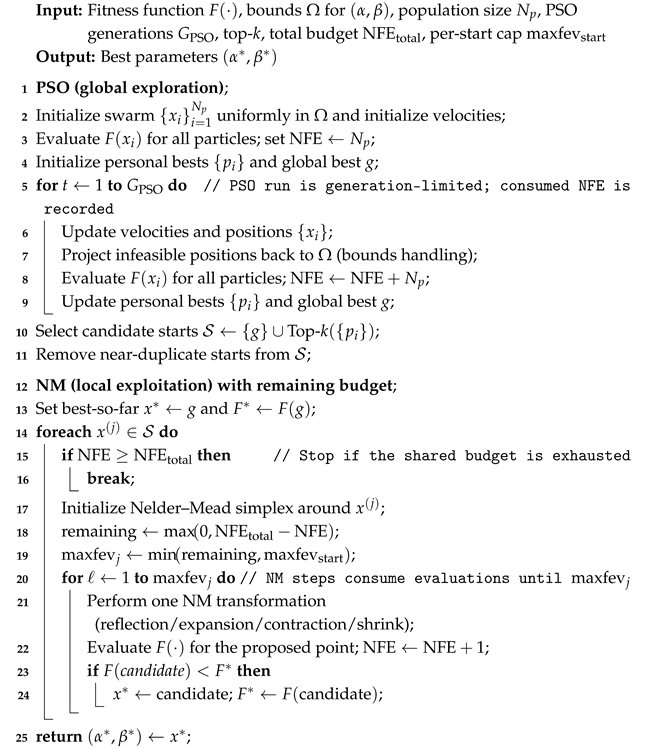


##### Computational Complexity Analysis

The computational complexity of the proposed BC-PSO-NM framework can be analyzed by considering the cost of its two main stages. For the PSO phase, the complexity can be approximated as

$$O(N_{p}\times G\times n),$$

Here, $N_{p}$ denotes the swarm size, *G* represents the number of generations, and *n* is the dimensionality of the search space. This complexity arises from evaluating $N_{p}$ candidate solutions at each generation, where the objective function depends on *n* variables.

The NM refinement stage operates on a reduced set of promising solutions obtained from the PSO phase. The computational cost of the NM algorithm is associated with simplex operations in the *n*-dimensional search space. However, in the proposed framework, the number of evaluations performed by NM is strictly limited by the remaining function evaluation budget.

Therefore, the total computational cost of the hybrid framework is bounded by the predefined evaluation budget ${\mathrm{NFE}}_{\mathrm{total}}$, which ensures a controlled balance between global exploration and local exploitation while maintaining computational efficiency.

#### 4.4.3. Configuration of Comparative Algorithms

To assess the efficiency of the proposed BC-PSO-NM optimization algorithm, it was compared with commonly used bio-inspired optimization algorithms such as PSO and its variants (SPSO and constriction PSO), DE, GA, and ABC. To provide a clean and representative comparison, well-established algorithms from different metaheuristic families commonly applied to image processing were selected. For example, PSO and ABC belong to swarm intelligence optimization methods, whereas GA and DE represent classical evolutionary algorithms, both applicable to continuous optimization, with DE being particularly well-suited for real-valued search spaces. By selecting representative algorithms from different metaheuristic families, the experimental design enables a balanced assessment of the proposed hybrid method across distinct search paradigms.

All algorithms were configured with a population size of $N_{p}=20$ and executed under the same total NFE budget. Based on preliminary convergence analysis, fitness stabilization was observed around 22 iterations. Therefore, the total NFE budget (${\mathrm{NFE}}_{\mathrm{total}}=440$) was fixed uniformly across all methods to ensure sufficient convergence while avoiding unnecessary computational overhead.

The parameter settings were adopted from configurations previously reported in the literature for image contrast enhancement and related optimization tasks, where they have demonstrated stable convergence behavior and competitive performance [43,44,45]. This strategy reduces the influence of arbitrary parameter selection and facilitates reproducibility and fair comparison among algorithms. The parameters were defined as follows:

- PSO: acceleration coefficients $c_{1}=c_{2}=1.7$;
- SPSO: inertia weight w=0.7289, acceleration coefficient $c_{1}=c_{2}=1.7$;
- Constriction PSO: acceleration coefficient $c_{1}=c_{2}=2.05$;
- DE: crossover probability CR=0.5, differential weight F=0.9;
- GA: crossover probability 0.9 with distribution index $\eta_{c}=15$; mutation; probability 0.5 with distribution index $\eta_{m}=20$;
- ABC: scout abandonment limit scaling factor 0.5.

### 4.5. Radon Transform and Sinogram Generation

Sinograms were directly generated using the Radon transform, ensuring consistency with the physical image formation model. To ensure reproducibility of the experiments, sinograms were generated using a fixed acquisition configuration consisting of:

- Number of projection angles: 180;
- Number of detector elements: 224;
- Resulting sinogram size: 224×180.

For each projection angle, the Radon transform was computed by integrating image intensities along lines oriented at the specified angle, simulating X-ray projections. The set of discrete projection profiles obtained across all angles forms the final sinogram representation.

The use of 180 projection angles provides one-degree angular resolution, ensuring sufficient angular sampling while maintaining computational efficiency during optimization. After generation, each sinogram was normalized to a consistent intensity range to avoid scaling bias across samples and to promote stable convergence of the bio-inspired algorithms.

### 4.6. Classification of Enhanced Sinograms

To evaluate the clinical relevance of the proposed contrast enhancement framework, the enhanced sinograms were subsequently used for lesion classification into benign and malignant categories. Importantly, the classification stage was performed directly on the enhanced sinograms, without any reconstruction step, reinforcing the hypothesis that diagnostically relevant information can be preserved and exploited in the projection domain.

A Vision Transformer (ViT-B/16) pretrained on ImageNet was selected as the teacher model due to its ability to model long-range contextual relationships through self-attention mechanisms and its demonstrated effectiveness across a broad range of medical image analysis tasks [46]. MobileNetV2 pretrained on ImageNet served as the student model due to its lightweight architecture and suitability for knowledge distillation.

All images were resized to 224×224 and normalized using ImageNet statistics (mean = [0.485, 0.456, 0.406], std = [0.229, 0.224, 0.225]). Data augmentation was applied exclusively to the training subset to improve generalization and reduce overfitting. The augmentation pipeline included random horizontal flipping, slight intensity variations (brightness and contrast), and minor geometric transformations (small rotations, translations, and scaling). No augmentation was applied to the validation or test sets to ensure an unbiased evaluation. The dataset was split using stratified sampling into 70% training, 10% validation, and 20% testing sets. To ensure reproducibility, a fixed seed (42) was used for Python (version 3.10.18), NumPy (version 2.2.6), and PyTorch (version 2.9.1), and deterministic CUDA behavior was enabled.

To address class imbalance and improve robustness, a class-balanced resampling strategy with replacement was applied exclusively to the training set. This procedure ensures that both classes are equally represented during training and can be interpreted as a bootstrap-like mechanism that enhances stability under limited and imbalanced data conditions. In addition, weighted cross-entropy was employed, where class weights were computed using inverse frequency normalization. The student model was trained using the Adam optimizer with a learning rate $1\times 10^{-4}$, a batch size of 32, and a maximum of 50 epochs. Knowledge distillation was implemented using the Joint Graph-based Equilibrium Knowledge Distillation (JGEKD) loss, combined with cross-entropy as:

(17) $$L=L_{CE}+\lambda \;L_{KD},$$

with λ=0.5. Early stopping was applied with a patience of 5 epochs based on the validation F1-score of the malignant class, and the best-performing student checkpoint was retained for final testing.

For a fair comparison, classification experiments were conducted using sinograms generated by all enhancement methods, including a traditional linear intensity adjustment baseline, PSO and its variants (SPSO and Constriction PSO), DE, GA, ABC, and the proposed BC-PSO-NM algorithm. Classification performance was assessed using standard metrics, including Precision, Recall, and F1-score, reported separately for benign and malignant classes. In addition, confusion matrices were analyzed to examine class-wise error distributions and to identify potential bias toward false positives or false negatives.

## 5. Results

This section presents the experimental results obtained from the proposed bio-inspired contrast enhancement framework. The evaluation focuses on five complementary aspects: (i) convergence behavior under a fixed NFE budget, (ii) quantitative image quality assessment, (iii) qualitative visual analysis, (iv) algorithm execution time, and (v) downstream lesion classification performance in the projection domain.

For reproducibility, the proposed BC-PSO-NM framework was implemented in Python (version 3.10.18) using widely adopted scientific computing libraries. The optimization components were developed using the PyMOO library (version 0.6.1.5) together with BeeColPy (version 2.3.2) for the implementation of bio-inspired optimization algorithms. Image processing operations, including sinogram generation and manipulation, were performed using OpenCV (version 4.12.0). The classification stage was implemented using the PyTorch deep learning framework (version 2.9.1). All experiments were conducted on a workstation equipped with a 13th-generation Intel Core i5 processor, 24 GB of RAM, and an NVIDIA RTX 5050 GPU, running Windows 11.

### 5.1. Convergence Analysis

**Figure 2:**
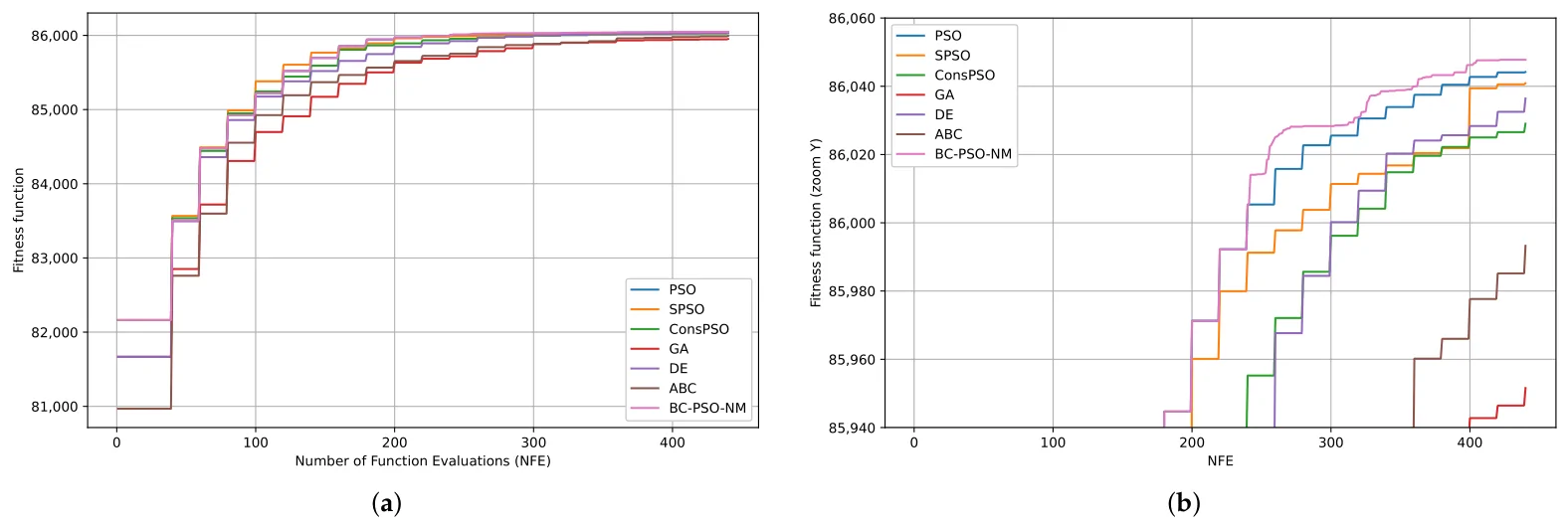
Convergence behavior of the evaluated bio-inspired algorithms. (**a**) Mean fitness versus NFE over the full evaluation budget. (**b**) Zoomed-in view of the convergence curves to highlight early-stage performance differences.

To evaluate optimization efficiency, the fitness evolution with respect to the NFE was recorded for all algorithms. To ensure reproducibility and fair stochastic comparison, p 13 representative images were evaluated across 22 independent runs. In each run, a distinct random seed was used, while the same seed was maintained across all algorithms to ensure identical initialization conditions. This design prevents bias due to stochastic initialization differences and enables controlled convergence comparison. The convergence curves shown in Figure 2 correspond to the mean fitness trajectory across all runs.

As observed in Figure 2a, the proposed BC-PSO-NM method achieves faster fitness improvement during the early stages of the optimization process. The zoomed view in Figure 2b highlights reduced oscillatory behavior and improved stability compared to standalone bio-inspired algorithms under the same evaluation budget. Notably, the hybrid algorithm reaches near-plateau fitness values with fewer evaluations, suggesting improved exploration–exploitation balance under constrained computational budgets. This behavior can be attributed to the transition from population-based exploration to deterministic local refinement, which accelerates convergence once promising regions of the search space have been identified.

### 5.2. Quantitative Image Evaluation

To quantitatively assess the impact of the proposed BC-PSO-NM algorithm on contrast enhancement performance, full-reference image quality metrics were evaluated under a fixed NFE budget. The analysis focuses on PSNR, SSIM, and FSIM, which respectively measure reconstruction fidelity, structural preservation, and perceptual feature similarity.

Based on the convergence behavior observed in Section 5.1, an additional experiment was conducted by limiting the optimization process to 15 iterations, enabling assessment of performance under reduced computational effort while maintaining competitive image quality. The results are summarized in Table 1, where the mean values and standard deviations are reported. The symbols “+”, “−”, and “≈” denote statistically significant improvement, degradation, or no significant difference, respectively, according to the Wilcoxon signed-rank test at α=0.05.

BC-PSO-NM achieved the best mean PSNR and SSIM values and remained statistically equivalent to PSO, SPSO, and Constriction PSO in most comparisons. In contrast, DE, GA, ABC, and the linear adjustment baseline exhibited significantly lower performance in multiple metrics according to the Wilcoxon signed-rank test. These results indicate that the proposed hybrid framework consistently matches or surpasses the performance of standalone bio-inspired optimizers while maintaining competitive image quality under the same NFE budget.

Although the linear adjustment baseline achieved the highest mean FSIM value, it exhibited substantially lower PSNR and SSIM values, indicating that improvements in perceptual similarity alone do not necessarily translate into better reconstruction fidelity or structural preservation. This observation suggests that perceptual similarity alone is insufficient to characterize enhancement quality in projection-domain breast imaging, where structural preservation and downstream diagnostic performance are also critical considerations.

### 5.3. Qualitative Image Evaluation

To facilitate qualitative assessment, visual comparisons are organized in two complementary domains. First, contrast-enhanced mammograms are presented to analyze spatial-domain improvements in local contrast and structural visibility. Subsequently, the corresponding sinograms generated via the Radon transform are displayed to evaluate the preservation of projection-domain patterns and angular coherence. This dual-domain visualization enables assessment of enhancement consistency across transformations while examining potential over-amplification or structural distortion effects.

**Figure 3:**
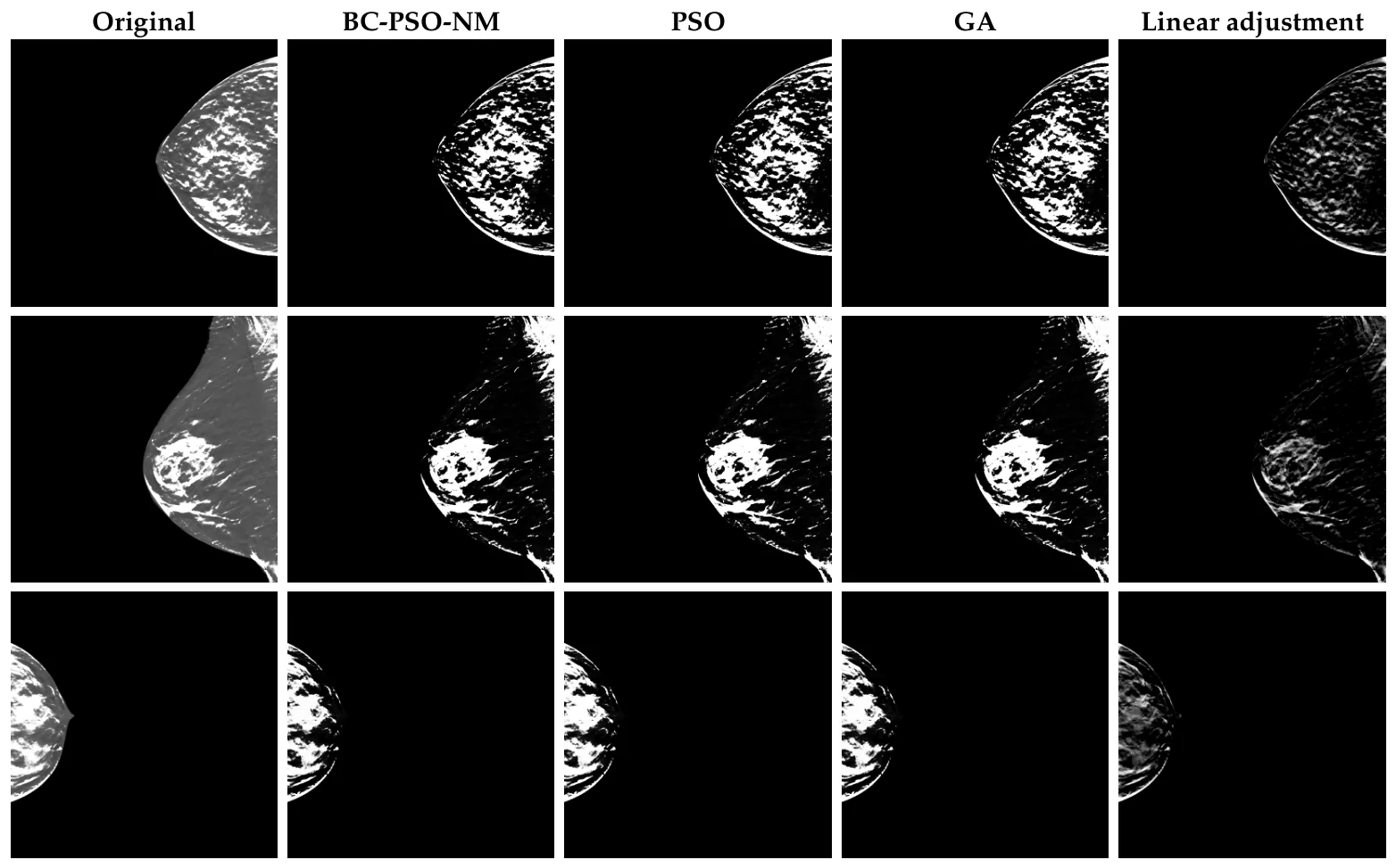
Representative visual comparison of mammogram contrast enhancement using the original image, BC-PSO-NM, PSO, GA, and linear adjustment. Rows correspond to representative cases with low, average, and high image quality metrics.

**Figure 4:**
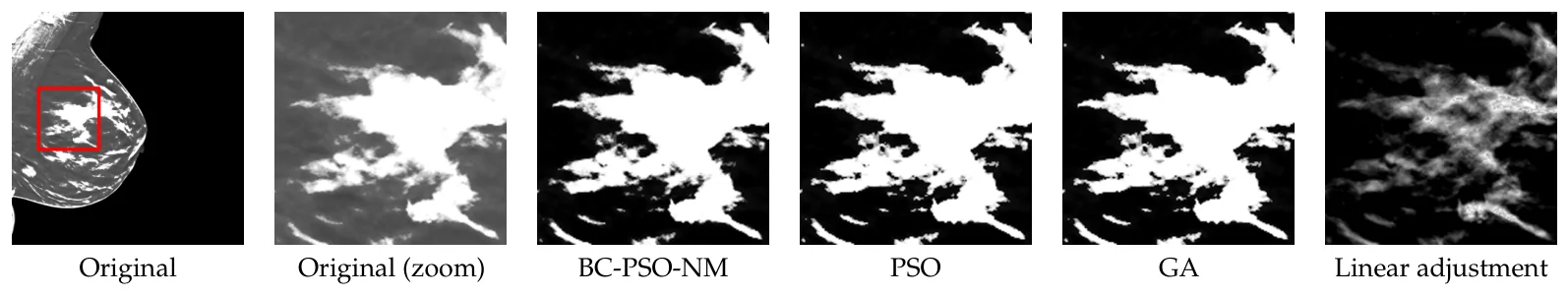
Visual analysis of a representative lesion case. The first column shows the original mammogram, where the red box indicates the selected ROI. The second column presents the corresponding zoomed-in region, followed by contrast enhancement results using BC-PSO-NM, PSO, GA, and linear adjustment.

Figure 3 illustrates the contrast enhancement results obtained using the evaluated methods. To avoid visual clutter and improve interpretability, only selected approaches are shown, including the original image, the proposed BC-PSO-NM method, a representative bio-inspired optimization algorithm (PSO), a classical evolutionary algorithm (GA), and a traditional linear adjustment baseline. To provide a more detailed visual assessment, a zoomed-in analysis of a representative lesion region is presented in Figure 4. The region of interest is highlighted in the original image, and the corresponding zoomed-in comparison allows a clearer inspection of local contrast differences. Although visually subtle, these differences align with the observed improvements in quantitative metrics.

From the spatial-domain perspective, all optimization-based methods enhance contrast within the defined region of interest while preserving the global anatomical structure. Compared to linear intensity adjustment, the bio-inspired approaches exhibit improved boundary delineation and reduced background amplification. Visual differences among advanced optimization strategies are subtle; however, the proposed BC-PSO-NM method demonstrates consistent intensity redistribution across cases with different quantitative behaviors, which can be seen in detail in Figure 4.

**Figure 5:**
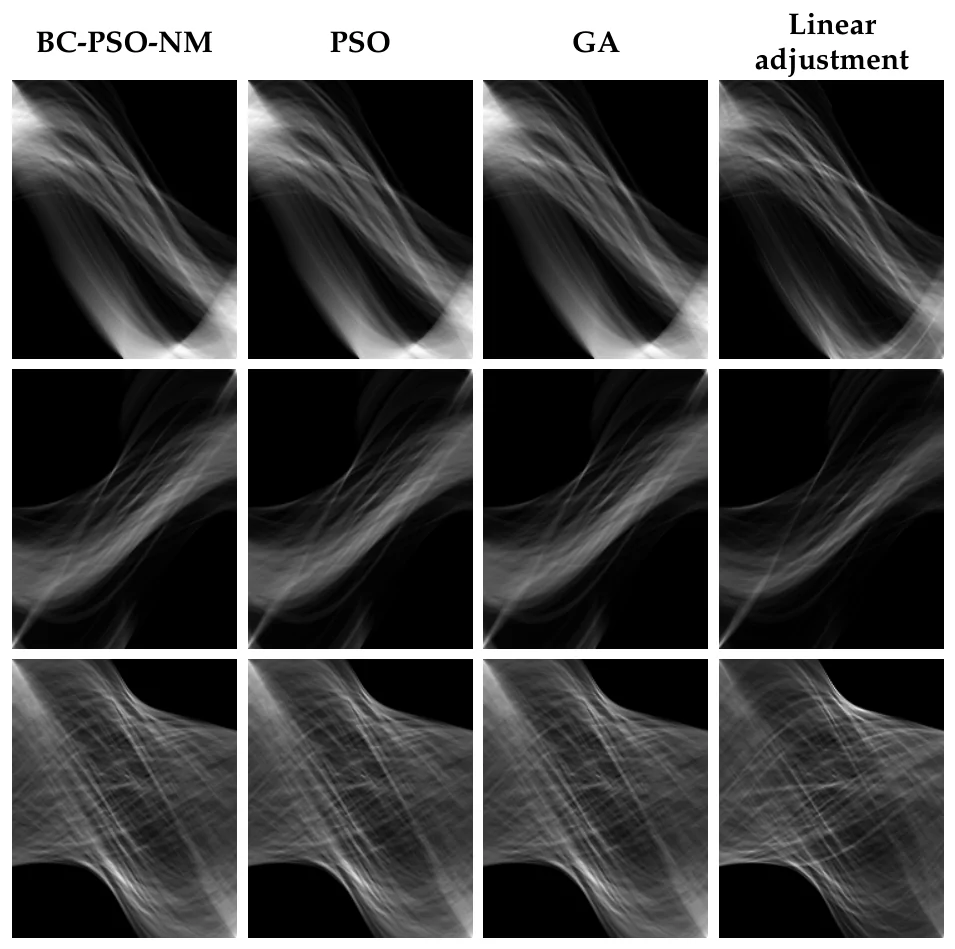
Representative comparison of projection-domain sinograms generated from mammograms enhanced using BC-PSO-NM, PSO, GA, and linear adjustment.

Figure 5 presents representative projection-domain counterparts of the enhanced mammograms using a subset of the evaluated methods. These sinograms allow evaluation of angular continuity and intensity redistribution after transformation. To improve visual clarity, only selected approaches are shown, including the proposed BC-PSO-NM method, representative bio-inspired optimization and evolutionary algorithms (PSO and GA), and a traditional linear adjustment baseline. Notably, the hybrid approach preserves coherent projection trajectories while enhancing contrast in diagnostically relevant regions.

### 5.4. Execution Time Evaluation

While visual and quantitative analyses demonstrate consistent contrast improvement, computational efficiency remains a critical factor for practical deployment in clinical workflows. To further evaluate the practical impact of the proposed hybrid framework, Table 2 compares the average execution time per image across all evaluated optimization methods. The reported times correspond to the complete contrast enhancement process. In addition, a comparison with a previously reported state-of-the-art method for sinogram contrast enhancement is included to further validate the computational efficiency of the proposed approach in a broader research context. While traditional linear adjustment remains computationally minimal, it lacks adaptive optimization capability.

The results show that the proposed BC-PSO-NM framework requires an average of 5.687 s per image. Of this runtime, 4.633 s correspond to the PSO exploration stage, while 1.054 s correspond to the Nelder–Mead refinement stage. This breakdown shows that most of the computational effort is associated with the global exploration phase, while the local refinement stage contributes only a small additional overhead. The proposed BC-PSO-NM framework required 5.687 s per image, representing a reduction of more than 97% compared to the PSO-based method reported in [5]. This improvement is primarily attributed to the explicit NFE budget constraint and the allocation of evaluations between global exploration and local refinement.

### 5.5. Classification Performance

**Figure 6:**
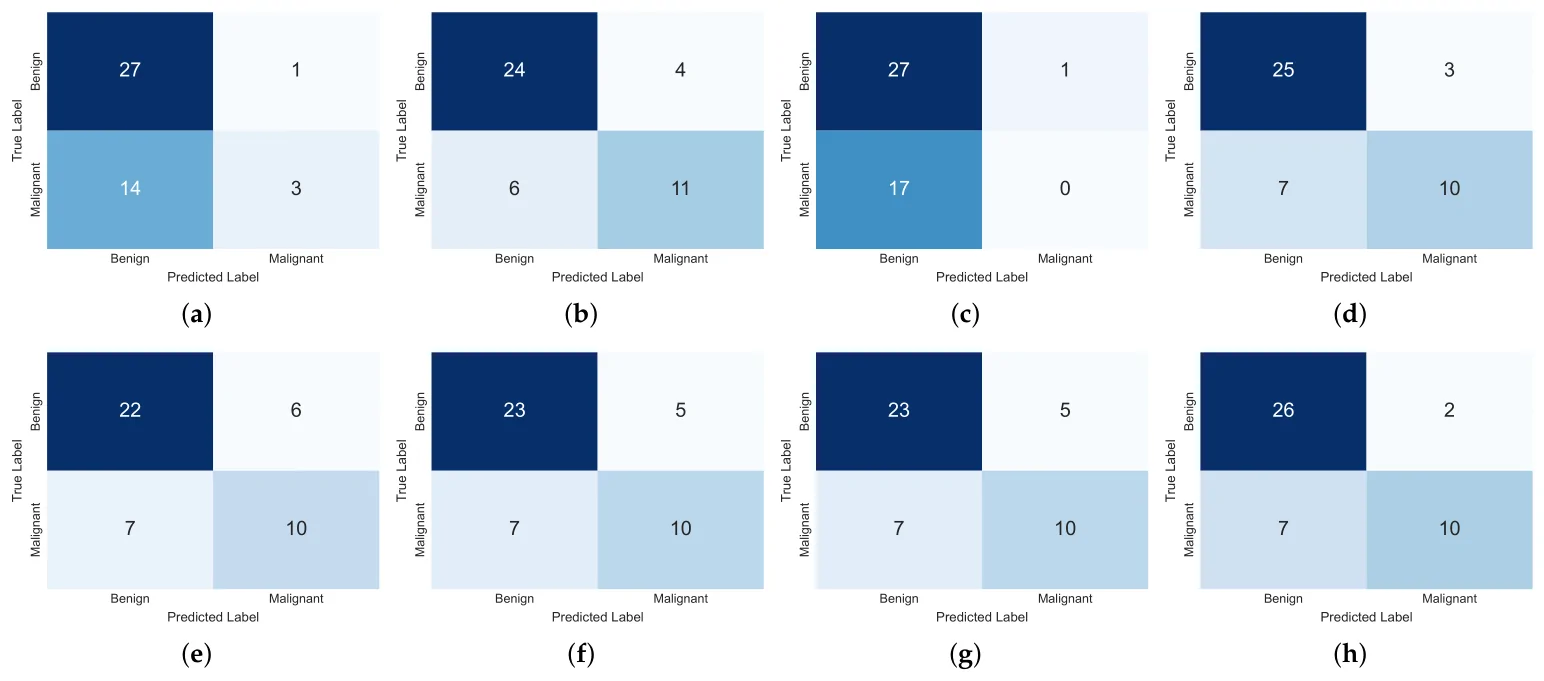
Confusion matrices for lesion classification across all evaluated enhancement methods: (**a**) Linear Adjustment, (**b**) PSO, (**c**) SPSO, (**d**) Constriction PSO, (**e**) GA, (**f**) DE, (**g**) ABC, and (**h**) the proposed BC-PSO-NM method.

To evaluate the clinical relevance of the proposed enhancement strategy, lesion classification was performed directly on the enhanced sinograms without reconstruction. Table 3 summarizes the quantitative classification metrics for all evaluated enhancement methods, while Figure 6 presents the corresponding confusion matrices, providing a detailed view of class-wise performance and highlighting differences in classification behavior across enhancement strategies. To provide a more compact summary of overall performance, Table 4 reports global evaluation metrics, including Accuracy, Balanced Accuracy, and Macro F1-score. These metrics offer a holistic view of classification behavior, particularly in the presence of class imbalance.

Although differences in PSNR, SSIM, and FSIM were relatively small across several optimization methods, more noticeable differences emerged in the downstream classification task. The classification results show that the proposed BC-PSO-NM method achieves the highest F1-score for the benign class and remains among the top-performing methods for the malignant class. While some competing approaches achieve strong performance in specific metrics, they exhibit imbalanced behavior across classes. For example, SPSO fails to correctly classify malignant samples, and linear adjustment shows very low recall for the malignant class. The confusion matrices further illustrate these differences in classification behavior across methods. BC-PSO-NM correctly classified 26 of 28 benign cases and 10 of 17 malignant cases. In comparison, PSO correctly identified 24 benign and 11 malignant cases, whereas SPSO failed to correctly classify any malignant sample. Linear adjustment also showed a marked bias toward the benign class, correctly identifying only 3 of 17 malignant cases. As shown in Table 4, the proposed BC-PSO-NM method achieves the best overall performance across all global metrics, obtaining the highest Accuracy and Macro F1-score, and matching the best Balanced Accuracy. This confirms that the proposed enhancement strategy not only improves class-wise performance but also leads to more consistent and reliable classification outcomes when considering the dataset as a whole.

These results indicate that hybrid contrast optimization enhances lesion separability in the projection domain and promotes a more balanced classification behavior across classes compared with several competing enhancement methods.

## 6. Discussion

This section discusses the implications of the experimental findings from both optimization and classification perspectives. First, the performance of the proposed hybrid framework is analyzed in terms of convergence behavior, image quality, and computational efficiency. Subsequently, the impact of contrast enhancement on downstream lesion classification is examined, followed by a discussion of the broader implications for projection-domain image analysis. Finally, the main limitations of the study and potential directions for future research are presented.

### 6.1. Performance of the Proposed Hybrid Framework

The experimental results demonstrate that the proposed BC-PSO-NM framework provides a consistent balance between convergence efficiency, image quality preservation, and downstream classification performance. From a biomimetic perspective, the method follows a high-level relay hybridization strategy according to Talbi’s taxonomy, where PSO performs global exploration and Nelder–Mead subsequently refines promising solutions through local exploitation. This combination is particularly relevant for sinogram optimization, where the search space is highly structured and sensitive to local variations. Unlike conventional image-domain enhancement, projection-domain optimization must preserve angular continuity and projection consistency, as excessive local modifications may alter diagnostically relevant patterns encoded in the sinogram representation. By operating under a fixed NFE budget, the proposed framework achieves comparable or improved enhancement quality without increasing computational requirements, resulting in faster convergence and competitive execution times. Therefore, the proposed approach prioritizes convergence efficiency, stability, and scalability over marginal metric inflation, aligning with Green AI principles and resource-aware medical imaging systems. Rather than proposing a new hybrid metaheuristic, the main contribution of this work lies in its domain-specific application to projection-domain breast imaging under a controlled evaluation-budget scheme, demonstrating how the interaction between exploration and refinement can improve downstream classification performance.

Although the improvements observed in PSNR, SSIM, and FSIM are numerically modest, the Wilcoxon signed-rank test indicates that several of these differences are statistically significant. In particular, BC-PSO-NM consistently outperformed Constriction PSO, DE, GA, and ABC, while remaining statistically comparable to PSO and SPSO in most comparisons. These findings suggest that even small improvements in contrast distribution and structural preservation may influence the discriminative information available for downstream analysis.

### 6.2. Impact on Downstream Classification

This observation becomes more evident in the classification results. While several enhancement methods achieved similar image quality metrics, larger differences emerged in the downstream classification task. The proposed BC-PSO-NM framework achieved the highest overall Accuracy and Macro F1-score among the evaluated methods while exhibiting more balanced performance across benign and malignant classes than several competing approaches. In contrast, some competing methods exhibited unstable behavior despite competitive image quality metrics. For example, SPSO produced competitive enhancement metrics but failed to correctly identify malignant samples, indicating that improvements in conventional image quality measures do not necessarily translate into improved diagnostic discrimination.

These findings support the hypothesis that projection-domain contrast optimization is not merely a visual enhancement step but a functional preprocessing mechanism that can influence diagnostic performance. By preserving angular consistency while enhancing diagnostically relevant structures, the proposed framework appears to facilitate more informative feature representations for downstream classifiers. Furthermore, the method achieved these improvements while maintaining competitive execution times under a fixed evaluation budget, remaining substantially faster than previously reported sinogram enhancement approaches. This behavior aligns with Green AI principles and highlights the potential of resource-aware optimization strategies in medical imaging applications.

### 6.3. Implications for Projection-Domain Analysis

From a broader perspective, relatively few studies have investigated the joint relationship between contrast enhancement and classification directly in the sinogram domain. Therefore, the proposed framework contributes to the growing body of research on projection-domain image analysis by demonstrating that optimized sinograms can positively influence downstream classification performance. Although these findings should be interpreted cautiously due to the limited dataset size and the use of Radon-generated sinograms, they suggest that projection-domain enhancement may serve as a promising preprocessing strategy for future DBT-based computer-aided diagnosis systems.

### 6.4. Limitations and Future Research Directions

Despite these promising findings, two primary limitations should be acknowledged. Currently, publicly available datasets containing labeled DBT sinograms suitable for contrast enhancement and downstream classification studies are extremely limited or unavailable. Consequently, the sinograms analyzed in this work were generated using the Radon transform applied to labeled mammographic images. The Radon transform provides a well-established mathematical model of the tomographic projection process and preserves the fundamental geometric relationships of projection-domain imaging, enabling controlled experimentation while maintaining lesion annotations. Nevertheless, this representation does not fully reproduce system-specific acquisition characteristics such as detector response, acquisition noise, and angular sampling strategies. Therefore, future validation using raw DBT projection data would be valuable to further assess real-world clinical applicability.

A second limitation relates to the limited clinical availability of projection-domain data. Unlike reconstructed mammograms, sinograms are not routinely used for clinical interpretation, and radiologist-based assessment at this level remains largely unexplored. Consequently, the present study relies on quantitative image quality metrics and downstream classification performance as surrogate indicators of diagnostic relevance. This should not be interpreted as a limitation of the proposed framework itself, but rather as a current limitation of the broader research area, where projection-domain datasets and clinical evaluation protocols remain scarce.

Finally, although the experimental design incorporated multiple independent runs, controlled random seeds, and statistical significance testing to ensure robust comparisons, further validation on larger and more diverse datasets remains necessary to assess the generalizability of the proposed framework. Future research should therefore validate the proposed method using raw DBT projection data and larger multi-center datasets.

Overall, the results demonstrate that structured hybrid bio-inspired optimization in the projection domain can positively influence downstream classification performance, supporting the growing potential of sinogram-based analysis as a complementary direction for DBT computer-aided diagnosis systems. These findings further suggest that hybrid bio-inspired optimization can serve as an effective mechanism for balancing exploration and exploitation in projection-domain medical imaging applications under constrained computational budgets.

## 7. Conclusions

This study presented a Budget-Constrained Hybrid PSO–Nelder–Mead (BC-PSO-NM) framework for projection-domain contrast enhancement in digital breast tomosynthesis. By formulating contrast adjustment as a constrained parameter optimization problem under a fixed budget of 440 function evaluations, the proposed approach enables fair comparison among bio-inspired and evolutionary algorithms while explicitly controlling computational cost.

Experimental results demonstrate that the hybrid structure effectively balances global exploration and local exploitation, achieving accelerated convergence while maintaining competitive execution times compared with other bio-inspired and evolutionary algorithms, as well as previously reported PSO-based methods. The proposed method achieved the best mean PSNR and SSIM values and required an average execution time of 5.687 s per image, representing a reduction of more than 97% compared with the previous PSO-based approach.

The downstream classification results further demonstrated the relevance of the proposed enhancement strategy. BC-PSO-NM achieved an Accuracy of 0.80, a Balanced Accuracy of 0.76, and a Macro F1-score of 0.77, corresponding to the best overall classification performance among the evaluated methods. These findings support the hypothesis that projection-domain contrast enhancement is not only a visual refinement step but a functional component that can influence downstream lesion classification.

The main limitations of the present study include the use of sinograms generated through the Radon transform rather than raw DBT acquisition data, as well as the limited availability of publicly labeled breast tomosynthesis datasets. Future research will focus on validating the proposed framework using raw projection-domain DBT data acquired directly from tomosynthesis systems, as such data become available, and evaluating its performance on larger multi-center datasets. Additional directions include investigating its clinical applicability, testing adaptive ROI selection mechanisms, and analyzing the contribution of individual components within the fitness function to further improve generalization capability and optimization effectiveness.

## Figures and Tables

**Table 1:** Quantitative image quality comparison in terms of PSNR, SSIM, and FSIM (mean values with standard deviation in parentheses). The best mean value per metric is highlighted in bold. In addition, statistical significance was assessed using the Wilcoxon signed-rank test for paired samples at α=0.05, taking BC-PSO-NM as the reference method. The symbols indicate whether the compared method performs significantly better than (+), significantly worse than (−), or shows no statistically significant difference (≈) with respect to BC-PSO-NM.

	BC-PSO-NM	PSO	SPSO	Constriction PSO	DE	GA	ABC	Linear Adjustment
PSNR	**15.757 (1.759)**	15.752 ≈ (1.763)	15.751 ≈ (1.756)	15.735 ≈ (1.740)	15.709 − (1.728)	15.715 − (1.740)	15.711 − (1.744)	12.961 − (1.616)
SSIM	**0.753 (0.092)**	0.752 ≈ (0.092)	**0.753 ≈ (0.092)**	0.752 − (0.092)	0.751 ≈ (0.091)	0.751 − (0.092)	0.751 − (0.092)	0.702 − (0.105)
FSIM	0.911 (0.045)	0.910 ≈ (0.045)	0.911 ≈ (0.044)	0.910 − (0.045)	0.909 − (0.045)	0.909 − (0.045)	0.908 − (0.046)	**0.912 + (0.042)**
+/−/≈	–	0/0/3	0/0/3	0/2/1	0/2/1	0/3/0	0/3/0	1/2/0

**Table 2:** Runtime comparison in terms of average execution time per image under the same NFE budget across all evaluated optimization methods. The reported times correspond to the complete contrast enhancement process. A state-of-the-art sinogram contrast enhancement method is also included for reference. The best time is highlighted in bold.

Method	Runtime per Image (s)
BC-PSO-NM	5.687
PSO	5.843
SPSO	5.827
Constriction PSO	5.816
GA	5.841
DE	5.829
ABC	6.135
Linear Adjustment	**1.98**
PSO method proposed in [5]	210.57

**Table 3:** Classification performance using ViT distilled to MobileNetV2 across all evaluated enhancement methods. Metrics include Precision, Recall, and F1-score computed per class (benign and malignant). Results are obtained from classification on contrast-enhanced sinograms under identical experimental conditions. The best performance per metric is highlighted in bold.

Algorithm	Class	Precision	Recall	F1-Score
BC-PSO-NM	Benign	0.79	0.93	**0.85**
	Malignant	**0.83**	0.59	**0.69**
PSO	Benign	**0.80**	0.86	0.83
	Malignant	0.73	**0.65**	**0.69**
SPSO	Benign	0.61	**0.96**	0.75
	Malignant	0.00	0.00	0.00
Constriction PSO	Benign	0.78	0.89	0.83
	Malignant	0.77	0.59	0.67
GA	Benign	0.76	0.79	0.77
	Malignant	0.62	0.59	0.61
DE	Benign	0.77	0.82	0.79
	Malignant	0.67	0.59	0.62
ABC	Benign	0.77	0.82	0.79
	Malignant	0.67	0.59	0.62
Linear Adjustment	Benign	0.66	**0.96**	0.78
	Malignant	0.75	0.18	0.29

**Table 4:** Overall classification performance across different enhancement methods. Metrics include Accuracy, Balanced Accuracy, and Macro F1-score computed from classification on enhanced sinograms using a ViT distilled to MobileNetV2 under identical experimental conditions. The best performance per metric is highlighted in bold.

Algorithm	Accuracy	Balanced Accuracy	Macro F1
BC-PSO-NM	**0.80**	**0.76**	**0.77**
PSO	0.78	**0.76**	0.76
SPSO	0.60	0.48	0.38
Constriction PSO	0.78	0.74	0.75
GA	0.71	0.69	0.69
DE	0.73	0.71	0.71
ABC	0.73	0.71	0.71
Linear Adjustment	0.67	0.57	0.53

## Data Availability

Publicly available datasets were analyzed in this study. These data can be found at The Cancer Imaging Archive (TCIA), BCS-DBT collection, https://www.cancerimagingarchive.net/collection/breast-cancer-screening-dbt/ (accessed on 13 August 2026). The dataset description is available in [42].
